# Statistical software for analyzing the health effects of multiple concurrent exposures via Bayesian kernel machine regression

**DOI:** 10.1186/s12940-018-0413-y

**Published:** 2018-08-20

**Authors:** Jennifer F. Bobb, Birgit Claus Henn, Linda Valeri, Brent A. Coull

**Affiliations:** 1 Biostatistics Unit, Kaiser Permanente Washington Health Research Institute, 1730 Minor Ave #1600, Seattle, WA 98101 USA; 2 Department of Biostatistics, University of Washington, Seattle, WA USA; 3 Department of Environmental Health, Boston University School of Public Health, Boston, MA USA; 4 Psychiatric Biostatistics Laboratory, McLean Hospital, Belmont, MA USA; 5 Department of Biostatistics, Harvard T H Chan School of Public Health, Boston, MA USA

**Keywords:** Multiple exposures, Mixtures, Exposure-response, Variable selection, Health risk estimation

## Abstract

**Background:**

Estimating the health effects of multi-pollutant mixtures is of increasing interest in environmental epidemiology. Recently, a new approach for estimating the health effects of mixtures, Bayesian kernel machine regression (BKMR), has been developed. This method estimates the multivariable exposure-response function in a flexible and parsimonious way, conducts variable selection on the (potentially high-dimensional) vector of exposures, and allows for a grouped variable selection approach that can accommodate highly correlated exposures. However, the application of this novel method has been limited by a lack of available software, the need to derive interpretable output in a computationally efficient manner, and the inability to apply the method to non-continuous outcome variables.

**Methods:**

This paper addresses these limitations by (i) introducing an open-source software package in the R programming language, the *bkmr* R package, (ii) demonstrating methods for visualizing high-dimensional exposure-response functions, and for estimating scientifically relevant summaries, (iii) illustrating a probit regression implementation of BKMR for binary outcomes, and (iv) describing a fast version of BKMR that utilizes a Gaussian predictive process approach. All of the methods are illustrated using fully reproducible examples with the provided R code.

**Results:**

Applying the methods to a continuous outcome example illustrated the ability of the BKMR implementation to estimate the health effects of multi-pollutant mixtures in the context of a highly nonlinear, biologically-based dose-response function, and to estimate overall, single-exposure, and interactive health effects. The Gaussian predictive process method led to a substantial reduction in the runtime, without a major decrease in accuracy. In the setting of a larger number of exposures and a dichotomous outcome, the probit BKMR implementation was able to correctly identify the variables included in the exposure-response function and yielded interpretable quantities on the scale of a latent continuous outcome or on the scale of the outcome probability.

**Conclusions:**

This newly developed software, integrated suite of tools, and extended methodology makes BKMR accessible for use across a broad range of epidemiological applications in which multiple risk factors have complex effects on health.

## Background

Estimating the health effects of several concurrent exposures is of increasing interest in epidemiology. For example, in environmental health interest lies in estimating the impacts of multi-pollutant mixtures, such as air pollution [1], toxic waste [2], and persistent organic chemicals [3]. Although studies have traditionally focused on estimating the health impacts of individual exposures, it is increasingly being recognized that populations are exposed to a wide range of factors across multiple domains, including environmental stressors and genetic and psychosocial determinants, and that these factors should be considered in conjunction [4].

A major barrier to studying the joint effects of many exposures concurrently is the lack of established statistical methods and corresponding software. Estimating the health effects of environmental mixtures is challenging because (i) exposures often have nonlinear and non-additive (eg, interactive) relationships with health outcomes, (ii) a high-dimensional vector of exposures may lead to poorly fitting regression models as the number of exposures increases relative to the number of observations in the dataset, and (iii) exposures are often highly correlated. Additionally, there are often several objectives of a multi-exposure health effect analysis, which may include estimating the overall effect of the mixture, identifying individual components that are responsible for the health effects of the mixture, visualizing the exposure-response function, and detecting interactions among pollutants [5]. Several statistical methods have been proposed for estimating the health effects of multiple exposures, including machine learning methods such as random forests [6]; clustering methods and other dimensional reduction methods such as principal components analysis, factor analysis, and structural equation models; and regression penalization methods such as the lasso [7]. However, these methods have typically addressed some but not all of the challenges and/or scientific objectives described above. Reviews of prior methods and their limitations, as well as systematic comparisons of the performance of selected methods, have been published previously [1, 8–12].

Recently, we developed a new approach for estimating the joint health effects of multivariate exposures, Bayesian kernel machine regression (BKMR), that simultaneously addresses the challenges and scientific objectives described above [13]. First, through use of a kernel function, this approach estimates the multivariable exposure-response function in a flexible way that allows for nonlinear and non-additive effects, while adjusting for covariates including potential confounding factors. Second, the approach simultaneously incorporates variable selection on the (potentially large number of) exposures in a way that controls for multiple testing [14]; this enables a parsimonious representation of the exposure-response function. Third, we developed a hierarchical variable selection approach that addresses the issue of multicollinearity by first classifying highly correlated exposures into groups, and then simultaneously conducting variable selection on the groups of correlated exposures as well as on the individual exposures within each group. In our prior methodological work describing BKMR [13, 15], we conducted a comprehensive evaluation of the performance of this approach. Through simulation studies based on real-world datasets, we found that (i) BKMR could well estimate exposure-response functions that included both nonlinear and non-additive effects, (ii) BKMR could identify important mixture components through variable selection, and (iii) the hierarchical variable selection approach could detect important groups of highly correlated exposures even in situations where individual components could not be identified. Additionally, BKMR has been applied previously in both toxicological and epidemiological studies leading to scientific insights that were not uncovered using standard regression approaches [15, 16].

Several important gaps limit the applicability of statistical methods for estimating the health effects of multi-pollutant mixtures in environmental health studies. These include a lack of software applying new methodology, data-generating scenarios with complex features (e.g., clustered outcome data), and the need for computationally efficient algorithms that yield correct results in a fraction of the time. A particular challenge of studies estimating the health effects of mixtures is the need to visualize the high-dimensional exposure-response function and to conduct inference in the presence of possible nonlinear and interactive associations of the exposures with the health outcome.

Here we provide several contributions addressing these gaps. First, we introduce an open-source software package (*bkmr*) [17] that implements the new BKMR approach for studying mixtures within the R statistical program [18]. This software provides a general, user-friendly implementation of BKMR, along with a suite of functions for processing model output to enable investigators to address the multifold objectives of a multi-exposure heath effect analysis. Second, we demonstrate methods for characterizing high-dimensional exposure-response functions, including visualizing the exposure-response relationship, and estimating scientifically relevant summaries, such as overall, single-exposure, and interactive health effects. Third, we present extensions to BKMR to enable model fitting to a broader class of applications, including a probit regression implementation for binary outcomes, and the ability to include a random intercept to account for correlated outcome data. Finally, we illustrate how a fast version of BKMR that utilizes a Gaussian predictive process approach can substantially speed up the model fitting. All of the examples used to illustrate these methods are fully reproducible with the provided R code.

## Methods

### Overview of BKMR

We first provide a brief overview of BKMR. The kernel machine regression (KMR) model for a continuous outcome is given by

$$ {Y}_i=h\left({z}_{i1},\dots, {z}_{iM}\right)+{{\boldsymbol{x}}_i}^{\prime}\boldsymbol{\beta} +{\epsilon}_i, $$

where *Y*_*i*_ denotes the response for individual *i* (*i* = 1, …, *n*), *z*_*im*_ is the *m*^*th*^ exposure variable, *h* denotes the unknown exposure-response function to be estimated, ***β*** represents the effect of the covariates (note that ***x***_*i*_ is a vector), and the residuals *ϵ*_*i*_~*N*(0, *σ*^2^) are assumed to be independent and identically (iid) normally distributed with a common variance. As described below, the effect of the exposures of interest modeled through the *h* function is allowed to be nonlinear and non-additive; the effect of the covariates could be modeled either linearly or more flexibly (e.g., by specifying a spline basis with a fixed number of degrees of freedom [DF] for one or more covariates). Additionally, if any covariates are hypothesized to interact with components of the mixture, then those covariates may be also be included in *h*.

For studies of multi-pollutant mixtures, the function *h* may include a large number of exposures of interest, and the relationship between these exposures and the health outcome can be complex, including nonlinear associations of one or more exposures, as well as possible interactions. Even with just a few exposures in the mixture, the combination of nonlinear and non-additive associations can lead to a high-dimension exposure-response relationship. As an illustration, if one were to model each exposure in the mixture using a spline basis with three DF to allow for nonlinearity and also include all of the interaction terms, this would result in a model with 255 parameters in the case of 4 exposures, 1023 parameters in the case of 5 exposures, and more generally, (1 + *DF*)^*M*^ − 1 parameters in the case of *M* exposures. In this setting of a high-dimensional exposure-response function, it can be challenging to specify a set of basis functions (e.g., polynomial or spline terms), and fitting a model including all basis functions and their interactions, as illustrated above, can lead to problems with over-fitting. BKMR addresses this by using a kernel machine representation for *h*, which regularizes the high-dimensional exposure-response function (details are in [13]). Under the kernel machine representation, rather than directly model the association of the exposures with the health outcome, one instead specifies a kernel function *K*(***z***_*i*_, ***z***_*j*_) that induces correlation of health outcomes among individuals with similar exposure profiles ***z*** = (*z*_1_, …, *z*_*M*_). In particular, the KMR model assumes that two individuals with similar values of ***z*** (i.e. ***z***_*i*_ close to ***z***_*j*_) will have similar health risks (i.e., *h*_*i*_ = *h*(***z***_*i*_) will be close to *h*_*j*_ = *h*(***z***_*j*_)).

Operationally, by using the kernel machine representation, the KMR model may be expressed as a mixed-effect model [19], and within a Bayesian context, prior distributions are placed on all of the unknown parameters. The model is fit using Markov chain Monte Carlo (MCMC). Full details of the hybrid Gibbs/Metropolis-Hastings MCMC algorithm is described in Bobb et al. (2015) [13] and the supplemental material thereof.

#### Incorporating variable selection

To incorporate variable selection, the kernel function may be augmented with auxiliary variables (*r*_*m*_, for *m* = 1, …, *M*), such that when the auxiliary parameter is equal to zero, then the corresponding exposure variable is no longer included in the model (i.e., if *r*_*m*_ is equal to zero then exposure *z*_*m*_ is not selected). Fitting BKMR with component-wise variable selection yields estimates of the posterior inclusion probabilities, which provide measures of variable importance for each exposure. Alternatively, one can apply a hierarchical variable selection approach, in which groups of exposures are specified. In this latter scenario, BKMR estimates the posterior inclusion probability for each pollutant group, as well as posterior inclusion probabilities among pollutants within each group, given that the group was selected into the model. An example of when the hierarchical variable selection approach is useful is to address the issue of multicollinearity by placing highly correlated pollutants into the same group. This approach is evaluated and compared to the component-wise variable selection approach by Bobb et al. (2015) [13].

#### Extension to clustered outcome data

In the setting of correlated outcome data, through repeated measures within the same person, or through individuals clustered within families or communities, the BKMR model can be extended as *Y*_*ij*_ = *h*(*z*_*ij*1_, …, *z*_*ijM*_) + *b*_*i*_ ***+ x***_*ij*_^′^***β*** + *ϵ*_*ij*_, where *Y*_*ij*_ is the response for observation *j* within cluster (e.g., person) *i*, ${b}_i\sim N\left(0,{\tau}_b^2\right)$ is a random intercept and *ϵ*_*ij*_~*N*(0, *σ*^2^) are the residual error terms.

### Methods for characterizing the exposure-response function

From fitting the BKMR model, one obtains an estimate of the exposure-response function *h*, which may include nonlinear and non-additive associations. Unless there are very few mixture components, it is not possible to visualize the entire exposure-response function all at once. Therefore, tools are needed to visualize cross-sections of *h*. One example of a cross-section of interest is to visualize how a single exposure is related to the outcome when all of the other exposures are fixed to specific level (e.g., median value). Similarly, one could visualize the bivariate relationship of two exposures with the health outcome, while fixing all of the other exposures to a specific level. These cross sections and others can be visualized using the *bkmr* software package and are illustrated below.

In addition to visualizing the exposure-response relationship, inference may be conducted on scientifically relevant summaries of interest. Here we define three such summaries, which quantify overall, single-exposure, and interactive health effects. In particular, we define the *overall effect* as the change in the mean outcome when all of the exposures (*z*_1_, …, *z*_*M*_) are fixed at their 75th percentile as compared to when all of the exposures are fixed to their 25th percentile, with all of the covariates ***x*** held constant. With notation, this is given by ${\varDelta}_{tot}\left(25,75\right)=h\left({z}_1^{75},\dots, {z}_M^{75}\right)-h\left({z}_1^{25},\dots, {z}_M^{25}\right)$, where ${z}_m^p$ denotes the *p*th percentile of the *m*th exposure variable. A second quantity of interest is a *single-exposure effect*, which we define as the change in the mean outcome when a single exposure is fixed at its 75th percentile as compared to when it is fixed at its 25th percentile, when all of the other exposures are fixed at their median value and all of the covariates ***x*** are held constant. For example, for exposure *z*_1_, this is given by ${\varDelta}_1\left(25,75\ \right|50\Big)=h\left({z}_1^{75},{z}_2^{50},\dots, {z}_M^{50}\right)-h\left({z}_1^{25},{z}_2^{50},\dots, {z}_M^{50}\right)$, and for other exposures the quantity *Δ*_*m*_(25, 75 |50) is defined analogously. Quantifying potential interaction is often another major goal of a mixtures health effect analysis. To facilitate this, we define an *interactive effect* as the difference in the single-exposure health effect when all of the other exposures are fixed at their 75th percentile, as compared to when all of the other exposures are fixed at their 25th percentile, given by *Δ*_*m*_(25, 75 |75) − *Δ*_*m*_(25, 75 |25). We note that the choice here of using the 25th and 75th percentiles is illustrative; these values may be modified as desired, and the above summaries can be calculated using any choice of threshold. Within a Bayesian framework, inference on the parameters above is conducted by calculating posterior mean estimates and 95% credible intervals for any of the numerical summaries of interest. Inference on other functionals of *h* that set exposures to fixed values may be conducted analogously.

### Probit BKMR for binary outcomes

BKMR can be extended to binary outcomes via generalized linear modeling. For reasons of computational efficiency for Bayesian inference, we use probit, rather than logistic, regression. The probit BKMR model is given by

$$ {\varPhi}^{-1}\left({\mu}_i\right)=h\left({z}_{i1},\dots, {z}_{iM}\right)+{{\boldsymbol{x}}_i}^{\prime}\boldsymbol{\beta}, $$

where *Φ* is the cumulative distribution function (CDF) for the standard normal distribution (*Φ*^−1^ is the probit link function) and *μ*_*i*_ = *P*(*Y*_*i*_ = 1) is the probability of an event (*Y*_*i*_ is a binary [0/1] variable).

It is well known that the probit model can be expressed using a latent normal random variable formulation. In particular, the probit model above can be expressed as ${Y}_i^{\ast }=h\left({z}_{i1},\dots, {z}_{iM}\right)+{{\boldsymbol{x}}_i}^{\prime}\boldsymbol{\beta} +{e}_i$, where *e*_*i*_ is standard normal and ${Y}_i=I\left({Y}_i^{\ast }>0\right)$ is equal to 1 if ${Y}_i^{\ast }>0$ and is equal to zero otherwise. Under this formulation, extension of the BKMR model from Gaussian outcomes to binary outcomes is relatively straightforward. One can simply apply the MCMC algorithm derived for normally distributed outcomes with an additional step of sampling from the posterior distribution of the latent ${Y}_i^{\ast }$ variables using a truncated normal distribution.

Although probit regression tends to be less common than logistic regression in many environmental health applications, it yields interpretable quantities both on the scale of the latent continuous outcome and on the scale of the outcome probability. In particular, by considering the latent normal formulation above, *h* may be interpreted as the relationship between the exposures and some underlying, continuous latent variable (Y^∗^). For example, if Y is an indicator variable for whether an individual has a particular health outcome, Y^∗^ could be interpreted as a latent marker of health status. Additionally, probit model coefficients can be converted into more familiar odds ratios using well-known formulae [20]. In particular, we have *logit*(*μ*) ≈ 1.6 · *Φ*^−1^(*μ*) so that *β*_*logit*_ ≈ 1.6 · *β*_*probit*_. This approximation works well provided the probability of the outcome event given the included predictors is not too close to 0 or 1.

### Gaussian predictive process

A major computational burden in fitting BKMR is the need to invert an n-by-n matrix (multiple times) at each iteration of the algorithm, where n is equal to the number of observations in the data. One way to reduce the computation time is to employ a Gaussian predictive process [21], an approach originally developed for large spatial datasets and which has been used previously in models with Gaussian process priors [22]. In this approach one specifies a set of points (referred to as ‘knots’) that covers the exposure space and then computes the projection of each vector of exposures onto the lower dimensional space spanned by the set of knots. Under this approximate approach, rather than needing to invert an n-by-n matrix, the algorithm only needs to invert a square matrix with dimension equal to the number of knots.

### Software implementation

The *bkmr* software is implemented as an R (R Development Core Team 2017) package. It has dependencies to the following packages: dplyr, magrittr, nlme, fields, truncnorm, tidyr, MASS, and tmvtnorm. The R software and these required packages can be obtained from the CRAN website at [https://cran.r-project.org/]. Furthermore, daily builds of package bkmr are provided on the CRAN website [https://cran.r-project.org/web/packages/bkmr/index.html]. It has been published under GPL version 2. Source code is available on GitHub at [https://github.com/jenfb/bkmr].

The package provides a complete framework for applying BKMR to conduct an analysis of the health effects of multiple exposures. The main function (*kmbayes*) implements a MCMC sampler to fit a BKMR model and includes the following features:

- the outcome may be either continuous or binary (specified using the *family* argument)
- option to include a random intercept to account for clustered or repeated measures outcome data (*id* argument)
- option to fit the model with or without variable selection (*varsel*)
- option to apply hierarchical variable selection (*groups*)
- implements a Gaussian predictive process approach to speed up model fitting for large sample sizes (*knots*)
- option to change default settings for the MCMC algorithm (*control.params* argument)

After fitting the BKMR model, a suite of post-processing functions are available, including functions to:

- provide a parsimonious summary of model output (*print* and *summary* methods)
- extract estimates of the posterior inclusion probabilities, which provide measures of variable importance for each exposure (*ExtractPIPs* function)
- extract summaries of posterior distributions of model parameters, including posterior mean, standard deviation, and quantiles (*ExtractEsts* function)
- obtain scientifically relevant summaries of the multivariable exposure-response function (these functions are illustrated in detail through the continuous outcome example below).

**Fig. 1:**
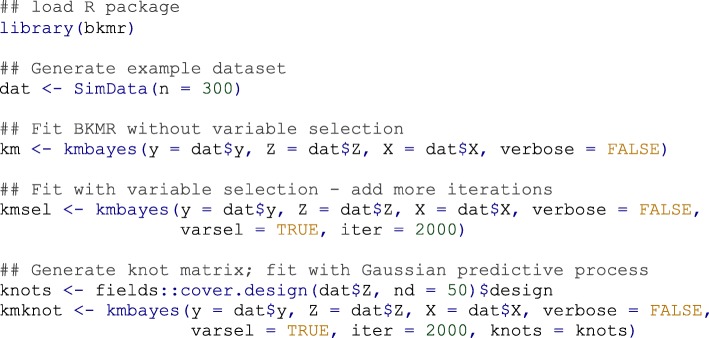
Usage example showing R code to fit BKMR with a continuous outcome. Here ‘y’ denotes the response vector of length *n* (where *n* is the number of observations); ‘Z’ is the *n*-by-*M* exposure matrix, where *M* is the number of exposure variables included in the exposure-response function *h*; and ‘X’ is the *n*-by-*P* covariate matrix, where *P* is the number of covariates

Example code illustrating the main kmbayes function is shown in Fig. 1. Additional details on the BKMR implementation are available in the package overview guide [https://jenfb.github.io/bkmr/overview.html].

### Practical considerations

Inference based on BKMR is contingent upon convergence of the MCMC algorithm. Several approaches can be used to monitor convergence, including visually inspecting the trace plots of model parameters, or more formal methods such as the Gelman-Rubin diagnostic [23]. The package overview guide [https://jenfb.github.io/bkmr/overview.html] provides details on how to modify the tuning parameters for running the MCMC algorithm in order to speed up convergence.

Additionally, it is good practice to evaluate the sensitivity of results to the choice of prior distribution specification. This can be done in the R package by changing the default settings. Of note, we have found that when conducting BKMR with variable selection, the magnitudes of the posterior inclusion probabilities can be sensitive to the choice of the prior distribution on the *r*_*m*_ parameters (though in our experience the relative ordering of the posterior inclusion probabilities has tended to remain stable) [15]. We therefore recommend varying the specifications of the prior distributions for these *r*_*m*_ parameters; additional guidance is given in the overview guide [https://jenfb.github.io/bkmr/overview.html], including an approach for incorporating prior knowledge on the degree of smoothness of the exposure-response function.

## Results

We illustrated the above approaches using two example datasets. For the first example, we applied BKMR to a simulated dataset that was generated as part of the 2015 workshop hosted by the National Institute for Environmental Health Sciences (NIEHS), titled “Statistical Approaches for Assessing Health Effects of Environmental Chemical Mixtures in Epidemiology Studies.” The goal of the workshop was to compare statistical methods by applying them to common datasets developed by epidemiologists and toxicologists based on real-world data applications [24]. A key feature of the workshop was that it used simulated datasets generated by scientists who did not develop the statistical methods being compared, which provides an objective benchmark for evaluating the methods’ performance. Applying BKMR in this setting illustrates the performance of the method in the context of a highly nonlinear, biologically-based dose-response function. For the second example, we considered a simulated dataset with a larger number of exposures and a dichotomous outcome, in order to illustrate probit BKMR.

### Continuous outcome example

For the continuous outcome setting, we used the first simulated data set (Data Set #1) from the NIEHS workshop [25], which included 7 exposure variables (*z*_1_, …, *z*_7_) and a single covariate *x* in 500 individuals. We applied BKMR to fit the model *E*[*Y*_*i*_] = *h*(*z*_*i*1_, …, *z*_*i*7_) + *βx*_*i*_, where *Y*_*i*_ denotes the response for individual *i*, *h* denotes the unknown exposure-response function to be estimated, and *β* represents the effect of the covariate. Reproducible code along with complete results from the analysis is available at [https://jenfb.github.io/bkmr/SimData1.html]; here we describe select results.

**Fig. 2:**
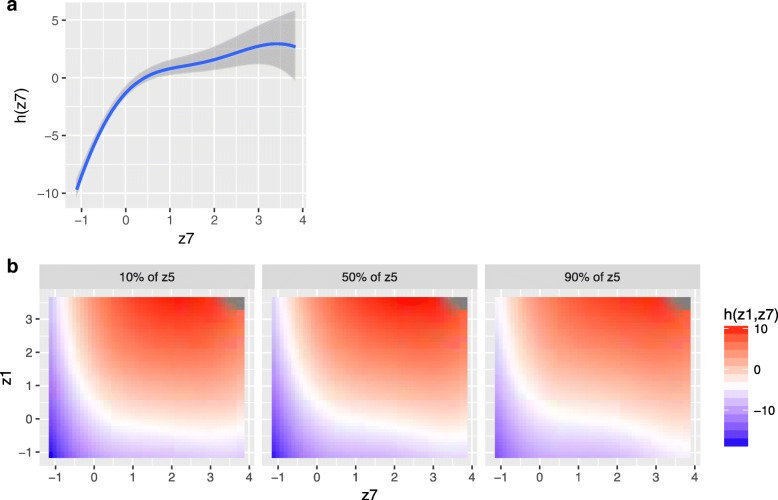
Cross-sections of the exposure-response function *h*(*z*_1_, …, *z*_7_), estimated using Bayesian kernel machine regression. **a** Univariate exposure-response function of *z*_7_ (95% credible intervals [CI]), where the remaining exposures are fixed at their median values. **b** Bivariate exposure-response function of *z*_7_ and *z*_1_ for *z*_5_ fixed at either its 10th, 50th, or 90th percentile, and for the remaining exposures fixed at their median values

As mentioned above, several functions are provided for processing the model output. Variable selection yields posterior inclusion probabilities (PIPs), whose values range from 0 to 1 and whose magnitude indicates relative variable importance. In the simulated example, the estimated PIPs were close to 0 for exposures *z*_3_ and *z*_6_ and were 1 for the remaining exposures. To illustrate the methods for visualizing the multivariable exposure-response function, we explored different cross-sections (Fig. 2). For example, Fig. 2a shows the (covariate-adjusted) association of *z*_7_ with the outcome, which indicates a nonlinear relationship with a steeper slope at lower levels of exposure that appears to plateau at higher exposure levels. Figure 2b shows the joint association of *z*_1_ and *z*_7_ with the response at different percentiles of a third exposure (*z*_5_), which is useful for visualizing potential three-way interactions, though in this example the similar pattern in association across levels of *z*_5_ suggests a lack of evidence of three-way interaction.

**Fig. 3:**
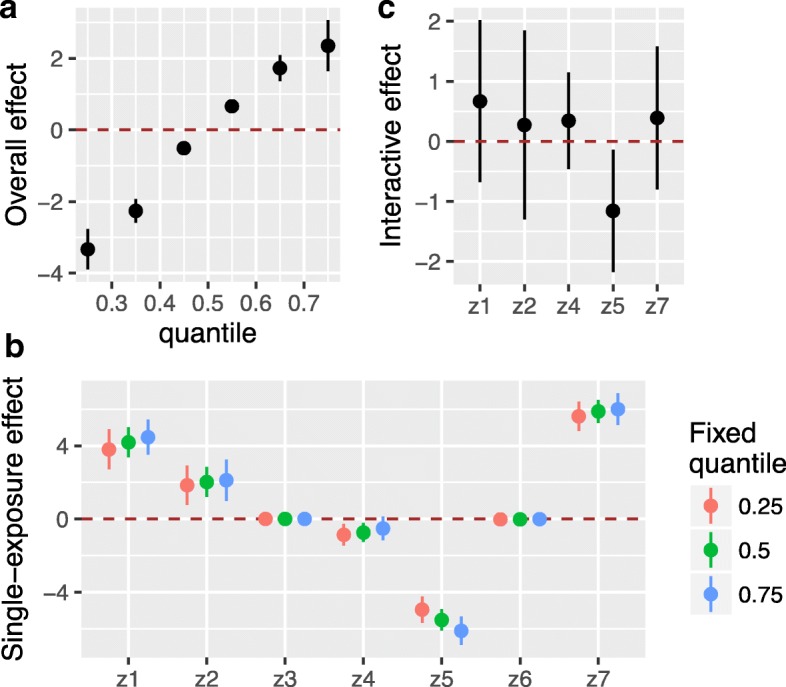
Numerical summaries of the exposure-response function *h*(*z*_1_, …, *z*_7_), estimated using Bayesian kernel machine regression. **a** Overall effect of the mixture (95% CI), defined as the difference in the response when all of the exposures are fixed at a specific quantile (ranging from 0.25 to 0.75), as compared to when all of the exposures are fixed at their median value. **b** Single-exposure health effects (95% CI), defined as the change in the response associated with a change in a particular exposure from its 25th to its 75th percentile, where all of the other exposures are fixed at a specific quantile (0.25, 0.50, or 0.75). **c** Interactive effects, defined as the change in the single-exposure health effects when all of the remaining exposures are fixed at their 25th percentile as compared to when they are fixed at their 75th percentile (i.e., red points from Panel **b** subtracted from the corresponding blue points)

We additionally calculated statistics summarizing the scientifically-relevant features of the exposure-response function described above (Fig. 3). Estimates of the overall effect of the mixture (3a) revealed that increasing levels of joint exposure were associated with higher levels of the outcome. To characterize the contribution of individual exposures to the overall effect, single-exposure effect estimates (3b) suggested that increases in exposure to *z*_7_, *z*_1_, and *z*_2_ were associated with higher levels of the outcome and that increases in exposure to *z*_5_ and *z*_4_ were associated with lower levels of the outcome. The single-exposure estimate for *z*_5_ was larger in magnitude when all of the remaining exposures were fixed at their 75th percentile as compared to when they were fixed at their 25th percentile, indicating possible interaction of *z*_5_ with one (or more) of the other exposure variables. To further explore this possibility, we calculated interactive effects (3c), which suggested that this interaction is statistically significant.

Comparisons of our results to the true exposure-response function used to generate the simulated dataset [26] demonstrate that BKMR was correctly able to identify which exposures were truly associated with the outcome and the direction of these associations. In addition, we were able to identify the nonlinear exposure-response relationship of the individual predictors, and to well-approximate the full exposure-response function that included both nonlinear and non-additive associations (See [https://jenfb.github.io/bkmr/SimData1.html]). Using the approximate, Gaussian predictive process method led to a reduction in the runtime of 49% when 100 knots were used (from 0.137 to 0.070 s per MCMC iteration) and a reduction in the runtime of 74% when 50 knots were used (to 0.036 s per iteration), without any substantial decrease in accuracy in estimating the exposure-response function in this example. Computations were performed using a 1.7 GHz processor with 8 GB of memory.

### Binary outcome example

**Fig. 4:**
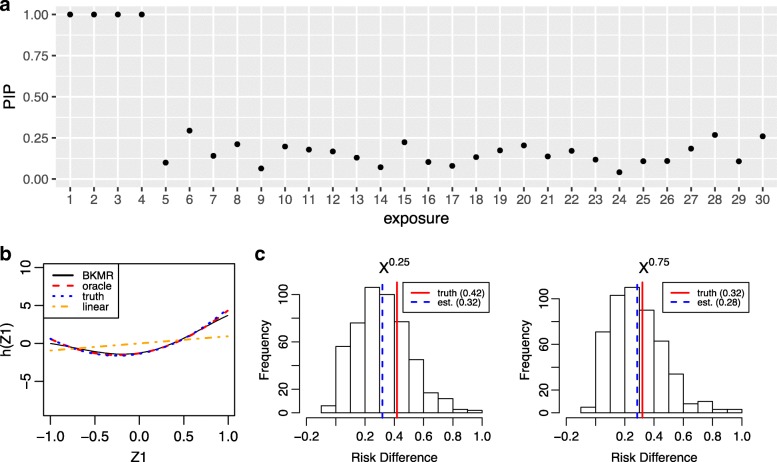
Example output from fitting probit Bayesian kernel machine regression to simulated data. **a** Posterior inclusion probabilities (PIPs) provide a measure of variable importance ranging from 0 to 1. Exposures 1–4 were included in *h* in the true data-generating model. **b** Univariate exposure-response function of *z*_1_ estimated from BKMR, in comparison to a probit generalized linear model (GLM) assuming linear terms of each of the exposure variables (“linear”), a probit GLM that uses the correct model form (“oracle”), and the true exposure-response function (“truth”). Under probit regression, *h* may be interpreted as the relationship between the exposure variables and an underlying, continuous latent outcome (e.g., a continuous marker of underlying health status for a binary health outcome). **c** Posterior distribution of the risk difference comparing the probability of the binary outcome when exposure 2 is at its 75th percentile versus its 50th percentile, for all of the exposures fixed at their median value, and for the single confounder *x* fixed at its 25th or 75th percentile (left and right panels, respectively), along with the posterior mean estimate (“est”) and the true risk difference (“truth”)

To illustrate probit BKMR, we simulated a dataset that included 30 exposure variables for a sample size of *n* = 200. The binary outcome depended on quadratic terms of four of the exposures and on a linear interaction term between two of these. Reproducible code and detailed results for this example are available at [https://jenfb.github.io/bkmr/ProbitEx.html]; select results are shown in Fig. 4. Posterior inclusion probabilities indicate that BKMR was correctly able to identify the variables included in the exposure-response function (4a) and to identify the quadratic exposure-response function without assuming this relation a priori (4b). As discussed above, the probit BKMR model yields interpretable quantities on the scale of the latent continuous outcome and on the scale of the outcome probability. Here, the u-shaped relationship of *z*_1_ suggests that both higher and lower levels of exposure may be associated with higher levels of the latent continuous outcome as compared to moderate levels of exposure.

We also illustrate how one can use the predicted probabilities from probit BKMR to compute quantities of interest, such as the risk difference (4c). For example, the point estimate (95% posterior credible interval) for the risk difference comparing the probability of the binary outcome when exposure 2 is at its 75th percentile versus its 50th percentile, for all of the remaining exposures fixed at their median value, was 0.42 (0.02, 0.73) when the single confounder *x* is fixed at its 25th percentile and was 0.32 (0.01, 0.72) when the confounder *x* is fixed at its 75th percentile. (The true risk difference was 0.32 and 0.28, respectively.) This indicates evidence of a statistically significant association between increasing levels of exposure 2 from moderate to high with an increased absolute risk of the outcome that persists across levels of confounder x.

## Discussion

The *bkmr* software package provides a general, open-source implementation of BKMR, a new and flexible approach for estimating the joint health effects of simultaneous exposure to multiple concurrent risk factors. The model specification can accommodate a broad range of data application scenarios common in environmental health, including continuous or binary outcomes, repeated-measures or clustered outcome data, and highly correlated exposures. A suite of functions is provided to process model output, addressing scientific questions of interest on features of the multivariate exposure-response relationship.

A key feature of BKMR is the estimation of the multivariable exposure-response function, which may often be high-dimensional in studies of the health effects of environmental mixtures. However, it can be challenging to conduct inference in this setting. Accordingly, we proposed several numerical summaries of the exposure-response function to allow investigators to estimate overall effects of the mixture, single-exposure health effects, and interactive effects. Rather than require quantities to correspond to specific parameters of a regression model (e.g., coefficients on main effect or interaction terms), as is often done in statistical modeling, the numerical summaries we proposed can be estimated regardless of the specific form of the regression model. Thus, they are broadly applicable to exposure-response relationships estimated from other (i.e., non-BKMR) statistical models. As with any dimension reduction technique, care is warranted when interpreting summary measures since they could mask potentially complex features of the data. For example, a single-exposure summary that compares health outcomes at high versus low exposure could appear null if there is a u-shaped relationship; likewise, an apparent null overall association may be observed if half of the exposures are positively associated and half are negatively associated with a similar magnitude. It is therefore recommended to explore a range of summary measures and to visualize different cross-sections of the exposure-response surface, together with the PIP, or variable importance, scores.

The MCMC algorithm we implemented for fitting BKMR employs several tricks to speed up the computation. First, rather than update the subject-specific effects of the mixture *h*_*i*_ within the main function used to fit BKMR, we marginalize the posterior distribution over these parameters [13]. These subject-specific effects are typically not themselves of scientific interest; rather, investigators often desire estimates of the general form of the exposure-response function, which can be visualized and summarized via the post-processing functions described above. Second, the implementation for binary outcomes utilizes the latent normal specification of probit regression, which has computational advantages for Bayesian inference. Third, the software allows for applying a Gaussian predictive process approach [21], originally developed for large spatial datasets, which projects the exposure space onto a smaller number of points (‘knots’), leading to efficient computation of health risk estimates.

Several additional functionalities could be added. Allowing for count outcome data via Poisson BKMR would enable the model to be applied to time series studies that estimate the joint health effects of day-to-day changes in multiple community-level risk factors (e.g., temperature and air pollution) on daily outcomes (e.g., hospitalization rates) [27]. However, implementation of the MCMC algorithm in this setting requires additional complexity because the computational tricks described above are not applicable. Additionally, the implementation focuses on a particular choice of kernel function for specifying the BKMR model, namely the Gaussian kernel. Our previous simulation studies showed that this specification is relatively flexible, accurately capturing a wide range of underlying forms of the true exposure-response function. However, the ability to specify other kernel functions could be added in the future. Along these same lines, our focus has been on estimating the joint health effects of continuous exposure variables; allowing for exposure-response surfaces that are functions of both categorical and continuous exposures may also be of interest. Finally, beyond estimating specific interactive-effect summary measures, one may be more broadly interested in detecting whether two groups of exposure variables interact [28–30]. This could be done within the BKMR framework by applying kernel decomposition methods to evaluate whether the kernel function h(**z**_**1**_, **z**_**2**_) could be expressed as h(**z**_**1**_) + h(**z**_**2**_) for two groups of exposures (**z**_**1**_ and **z**_**2**_).

## Conclusions

In summary, this newly developed software provides an integrated set of tools for conducting a mixtures health effect analysis. The software and expanded toolbox make BKMR accessible for use across a broad range of epidemiological applications in which a large number of exposures have complex, potentially nonlinear and interactive effects on health.

## Abbreviations

BKMR: Bayesian kernel machine regression
CI: Credible interval
DF: Degrees of freedom
KMR: Kernel machine regression
MCMC: Markov chain Monte Carlo
NIEHS: National Institute for Environmental Health Sciences
PIP: Posterior inclusion probability
